# Striving to establish patient participation in rehabilitation: the challenges experienced by nursing staff when changing practice to include the patient's perspective

**DOI:** 10.3389/fresc.2024.1504984

**Published:** 2024-12-19

**Authors:** Randi Steensgaard, Raymond Kolbaek, Helge Kasch, Sanne Angel

**Affiliations:** ^1^ Spinal Cord Injury Centre of Western Denmark, Viborg, Denmark; ^2^ Department of Public Health, Faculty of Health, Aarhus University, Aarhus, Denmark; ^3^ Department of Clinical Medicine - Neurology, Aarhus University, Aarhus, Denmark

**Keywords:** action research, nurses, rehabilitation, patient participation, spinal cord injury

## Abstract

**Purpose:**

Patient participation is a complex issue and difficult to establish, but essential to successful spinal cord injury rehabilitation. The purpose of this study was to explore the challenges experienced by nursing staff when they wanted to include the patient's perspective in their rehabilitation.

**Methods:**

Action research methodology was applied to increase knowledge, develop competences, and ultimately change practice. Over a period of two years, four nurses and four certified healthcare workers participated in identifying, testing and evaluating ways to improve patient participation. The data consist of evaluations of patient participation and recorded and transcribed dialogues from meetings and workshops. Transformed into text, data were analysed using the interpretive theory of Ricoeur to identify central themes.

**Findings:**

Three central themes highlight the challenges experienced by the nursing staff while focusing more on patient participation in nursing practice: (1) Struggling to really listen to the patient's perspective, (2) Searching for time to listen to the patient's perspective, (3) Legitimising the patient's perspective in rehabilitation.

**Conclusion:**

Even though nursing staff found patient participation to be vital for individualised rehabilitation, it was difficult to include the patient's perspective. The inclusion was challenged on a personal level, within the nursing group, and in the organisation due to prioritizing physical nursing tasks over the patient's perspective. Thus, the nursing staff struggled to change their nursing practice and to convince their colleagues and leaders that the rehabilitation should be re-organised to perform their nursing role to the full. This indicated a need to work on the attitude and approach of the entire organisation to promoting patient participation and nursing rehabilitation.

## 1 Introduction

The importance of patients' wishes, preferences and demands for providing high quality healthcare has become an agenda shared by politicians, health professionals and consumer organisations in Denmark and other Western European countries (1–4). Therefore, patient participation with this particular aim is central due to its many benefits such as increased quality of life, knowledge gained to make decisions on treatment, cost-effectiveness and a reduction in adverse events (1, 5–7).

Patient participation is especially important in rehabilitation after spinal cord injury (SCI) (8). Lindberg et al. found that rehabilitation, being structured around the individual patient's capacities, preferences and needs had implication for more personal tailored rehabilitation with constant considering of the patients varying ability to participate during the process of rehabilitation. However, this required that health professionals dedicated time and respectfully listened and paid attention to the patients' preferences and needs (8). The importance of patient participation in SCI rehabilitation is supported by several studies leading to patients taking control of their own lives, to autonomy and independence and to quality of life (9–11).

Unfortunately, participation is predominantly related to the observable physical performance and less to the person's subjective dimensions and inner perspective (12). However, being physical independent and self-sufficient may not always reflect the person's perspective and needs. Therefore, Van de Velde et al. (12) emphasise the need for health professionals to pay more attention to the patient's perspective. By engaging in dialogue, health professionals may reveal the patient's perspective, wishes and demands and tailor the rehabilitation process accordingly (10). This implies prioritising the nurse-patient relationship which is also highlighted in a review by Angel and Frederiksen (13) to be a key element to patient participation.

Even though patient participation is found to improve the quality of care (7, 11), and listening to the patient's perspective is a core element of nursing (14, 15), nurses still struggle to take the patient's perspective into account. Consequently, the patient's needs and wishes are not reflected in rehabilitation and care (16, 17). In addition, implementation efforts are inherently challenging and complex (18, 19). Therefore, the aim of the present study was to explore the challenges experienced by the nursing staff in their effort to improve patient participation in rehabilitation in a specific ward setting.

## 2 Methods

Due to known challenges of implementing patient participation in rehabilitation (18, 19) and the promising approach of involving health professionals (19–21) an action research study methodology was chosen with the involvement of nursing staff (22).

The action research design was based on activities including critical dialogue, reflection and action, and it was inspired by Dewey's philosophy and pragmatic approach (23, 24). The change-generating activities included workshops, meetings, developing, testing and the implementation of nursing initiatives to support patient participation. During these activities, nursing staff developed their skills and formed a common praxis in the group for reflection and building new knowledge (23, 25). The process followed four overall phases: (1) problem identification, (2) planning, (3) action, and (4) evaluation (26–28). The method emphasised that both failure and success provide learning opportunities and the possibility to correct the developed initiative or even dismiss it, if it proved ineffective (24, 29, 30).

### 2.1 Setting

The effort to improve patient participation in rehabilitation was made at the Spinal Cord Injury Centre of Western Denmark (SCIWDK) with a capacity of 35 beds and an out-patient clinic for life-long follow-up. Patients were hospitalized for 3–8 months during their initial rehabilitation sessions. Doctors, social workers, psychologists, occupational therapists and physiotherapists, nurses and social healthcare workers were all members of the inter-professional team providing rehabilitation. The centre serves a population of 3.5 million and is one of two highly specialized rehabilitation centres in Denmark.

### 2.2 Participants

Eight staff members, four registered nurses and four certified healthcare workers volunteered to participate in the study for two years (Supplementary Table S1 with alias names). The inclusion was based on their application of interest and motivation. All applicants were enrolled. They were named co-researchers, because they contributed actively with clinical expertise, knowledge, curiosity, reflection and drive (31, 32). The PhD supervisors participated in meetings and workshops on equal terms with the co-researchers and the PhD student who facilitated the processes.

To pave the way for organisational support of the study and of the co-researchers, an advisory board was established consisting of inter-professional managers (one doctor and one physiotherapist), PhD supervisors, two nursing managers, a patient representative (a former patient) and a co-researcher representative.

### 2.3 Data collection and preparation

Data on the challenges was collected in phase three; the co-researchers' experience of their effort to improve patient participation and phase four; their reflections, dialogues and evaluations. Hence, the data collected consisted of the co-researchers' written evaluations of their effort together with verbatim transcriptions of ten one-hour meetings and a one-day workshop conducted during the action and evaluation phases.

### 2.4 Analysis

In addition to the action research processes with immediate understanding during the actions, the written material produced an opportunity for in-depth analysis. We applied a phenomenological-hermeneutic analysis developed by Paul Ricoeur (33, 34). On three interrelated levels, the analysis moved from firstly an intuitive reading of the text, to secondly a disclosure of possible interpretations, and thirdly, a more universal understanding. At the first level, the naïve interpretation, we read the text several times to achieve an immediate understanding of the text as a whole. At the next level, the structural analysis, we read the text (sentence by sentence), working from what is said to an interpretation of what the text is about (34, 35), revelling central themes. Finally, at the third level, we conducted a critical interpretation of what was the most probable and significant understanding of what the text said about the nursing staff's challenges in establishing patient participation in rehabilitation. In a non-linear process, we went back and forth to develop a trustworthy interpretation of the text (34). This was further validated in the large material from all four phases for recognisability and finally related to current evidence on the subject.

## 3 Ethical considerations

The Danish Data Protection Agency approved the study (journal no. 1-16-02-503-15). The study was conducted in accordance with the Helsinki II Declaration (36) and the Danish Nursing ethical guidelines (37), and it received the approval of the SCIWDK interprofessional board.

The well-being of the co-researchers was a particular priority, as they openly shared thoughts, worries and insecurities to an extent requiring careful management. Hence, we made a written agreement with the regional clinic for occupational medicine and the psychologist at the centre to provide supervision and support if necessary.

## 4 Results

Working to improve patient participation the nursing staff experienced the value of eliciting the patient's perspective. Listening to the individual patient's needs, wishes and concerns, the nursing staff encouraged the patients to be open about thoughts on their situation. Doing so the patient's also shared experiences from their lives and their thoughts on how living with SCI would affect their future opportunities and position in society. A stronger relationship built on trust and honesty ensued from these conversations of depth and vulnerability, provided the patients with the time and place to take control and engage in the planning of their rehabilitation. Nevertheless, analysis showed that the profound positive experience of facilitating patient participation was challenged by obstacles which are presented in three central themes: (1) Struggling to really listen to the patient's perspective, (2) Searching for time to listen to the patient's perspective, and (3) Legitimising the patient's perspective in rehabilitation.

### 4.1 Struggling to really listen to the patient's perspective

The nursing staff struggled to set aside other tasks to make time for conversations. Their daily schedule of assisting patients with their personal routines was tight, as nursing staff had to ensure that patients were ready in time for training. Therefore, time to just listen to the individual patient to get to know the patient's perspective was either absent or minimal. The nursing staff were unaccustomed to spending time with their patients without performing practical tasks. Therefore, they struggled to be attentive to the patient's perspective and found it difficult to be sufficiently at ease to just listen:

‘We may well find this the most difficult thing of all. Because we are so used to collecting data. (…) and the fact that you just need to be there and must be able to deal with nobody saying anything for a while.’

(Co-researcher 2, workshop 3)

It was not only the pauses in the conversation that were difficult; the nursing staff also struggled with their urge to act while having conversations with the patients. One co-researcher mentioned that she would bring coffee to the conversation to appear calm and handle the unaccustomed situation. She said:

‘As nursing staff, we are trained, practiced and accustomed to acting, fixing, reacting, organizing, fussing—it is very rare that we just sit and talk.’

(Co-researcher 3, workshop)

Even though the nursing staff valued and acknowledged the benefit of the conversations, they struggled overcoming their reluctance. They felt they lacked training in conversation and felt a pressure to prioritize other practical tasks around the patient. Furthermore, they were needed elsewhere, and the thoughts of other tasks intruded on their concentration. Despite their difficulties, the nursing staff persevered and forced themselves to “sit on their hands” to make time and space for patient participation.

### 4.2 Searching for time to listen to the patient's perspective

The nursing staff felt compelled to prioritize other tasks on the ward rather than spending time with one patient for the purposes of having a conversation. As time was in short supply, the nursing staff often felt that they were letting down their colleagues:

‘..but it is difficult to get started on a conversation—when do we have the time? How many interruptions will we have? I feel bad leaving the ward. All those thoughts you have in your head when you go in and say that you will now be sitting with this patient for half an hour.’

(Co-researcher 4, workshop)

Conversations were viewed as something that could only take place when all other practical and administrative tasks on the ward were completed. Moreover, conversations were viewed as an add-on to rehabilitation, and they were expected to be set aside when other (physical or practical) tasks came up. In general, the nursing staff were expected to remain available for other tasks, and interruptions were commonplace accepted. The dilemma of wanting to live up to the expectations of colleagues and at the same time to facilitate patient participation became a source of frustration. The nursing staff became reluctant to plan and promise time dedicated to the individual patient, because they were unsure if they could keep their word:

‘..well, so I did not get to do that today. And that is probably what I find most stressful—all the good intentions. I became reluctant to make any appointments …’

(Co-researcher 1, meeting 16)

The nursing staff wanted to forge relationships with their patients built on trust, and cancelling appointments meant letting down their patients. Therefore, the nursing staff searched for alternative solutions and had the conversations with their patients within any setting, for example by the bedside, during other activities or in between practical activities, even though they knew from experience how important it was to have these conversations in calm settings with no other activities. An example was when the patient was seated on the toilet:

‘Co-researcher 5: (…) Many of my conversations take place in the bathroom. The patients relax and I perform the bowel management.

Co-researcher 4: Well, that was what we were talking about (…). Because it has actually been like that for many years, but it really ought not to be.

Co-researcher 5: You are absolutely right (…) I also think that we will probably have to; with all the fuss we have here regularly, we will have to grab the chance when we get it and not think about whether it is perfect …'

(Co-researchers 5 and 4, meeting 16)

The nursing staff ended up compromising their professional values and consequently seized the time around bowel management which is usually a private matter. Even though the other members of the staff opposed having conversations during intimate situations in the bathroom, they too described how they created space to elicit the patient's perspective in alternative ways. Accordingly, all nursing staff called for their care to include scheduled time to explore and establish the patient's perspective on more equal terms with other nursing activities.

### 4.3 The call for nursing to include the patient's perspective in rehabilitation

As undisturbed conversations were not a regularly occurring part of rehabilitation, the nursing staff felt they needed to argue for them as an integrated part of rehabilitation nursing. An example of the struggle and fight for legitimacy is voiced by one of the co-researchers:

‘..why is it not (…) equally valid to go in and say: we will have a conversation today; it is an integrated part of the treatment. You might just as well look at it like that …’

(Co-researcher 6, meeting 16)

The nursing staff emphasized the need to establish a mandate for conversation forming an essential aspect of the clinical pathway in line with other activities. This was further elaborated on in the next quote where the co-researcher describes the efforts needed to implement this approach in rehabilitation:

‘(…) it is part of the rehabilitation process here, it is part of the package, (…), why is it then that we have to put in so much effort to try to (…) get it changed instead of just being able to say to the patient: today (…) you and I are having a conversation …’

(Co-researcher 7, meeting 16)

The patient's scheduled routines and activities put pressure on the time nursing staff had with their patients. Therefore, they called for authority to plan the patient's time and to change nursing practice to include the patient's perspective in rehabilitation. However, the nursing staff did not have the mandate to accomplish that on their own.

## 5 Discussion

In the study, the nursing staff acknowledged how pivotal the patient's perspective was to patient participation and individualised care in rehabilitation. However, the findings show that despite the researchers and nursing staffs' effort to improve participation in the local setting, it was difficult for the nursing staff to find the time and space to listen to the patients calmly and attentively. There were identified difficulties on (1) a personal level, (2) within the nursing group, (3) the inter-professional team and (4) on the organisational level. The nursing staff struggled to change their nursing practice and convince their colleagues and leaders that the rehabilitation should be re-organisation to perform their nursing role to the full.

Numerous other studies find nurses struggle for a clear role, function and position in the inter-professional team (38–43). Even though many of the studies explore different elements of the nurse's role, (38–43), they do not provide answers neither as to how to prioritise in a busy rehabilitation ward nor how to embody new positions and functions. This is problematic because it may lead to a priority that does not support rehabilitation as it is intended. The idea of priority tasks (making the patient ready for training, preparing rounds, coordinating with the local authority, dispensing medicine, etc.) was a hindrance to conversation which was not defined as a task and therefore given low priority. This task-oriented culture is not solely confined to the rehabilitation centre in Denmark. Also Kitson and Athlin (44) argue that task-oriented nursing dominates nursing today in general, whereas building a relationship and encouraging patient participation are often omitted (44). Even though the nursing staff recognised the need to facilitate patient participation in rehabilitation, the pressure from task-oriented nursing activities impacted their time and calmness to establish a strong relationship and to elicit the patient's perspective, thereby impairing the function of rehabilitation nursing.

The unclear role and the low priority of tasks related to exploring the patient's perspective through conversation are accompanied by another obstacle that adds to the challenge for patient participation which is the patient's understanding of rehabilitation. Loft et al. (43) found a discrepancy between how the patients and the healthcare professionals value elements of rehabilitation and to some degree the patients view rehabilitation as equal to training. The fear of missing out on “real” rehabilitation equal to training led to a deprioritised rehabilitation pointing to increased self-care as Christiansen and Feiring (41) found in the context of stroke and brain injury rehabilitation. Patients in their study had difficulties understanding how morning care was more than merely something to get over and done with (41). This one-sided idea of rehabilitation being equal to training was also displayed in our study where the nursing staff took over at morning routines instead of actively involving the patient. Consequently, this led to an individual struggle for the nurses to do what they felt was needed to include the patient's perspective in rehabilitation on one hand and to live up to the patients' expectations and the organisations structure with scheduled training early in the morning.

To provide long-term improvement of patient participation, Elwyn et al. (18) emphasise the need to focus on the processes, interaction, impact on the healthcare professionals and the culture of the organisation when implementation strategies are evaluated. However, this study shows that even with local processes tailored to the setting and context, despite working with organisation and culture and an anchoring to the management in an advisory board, more drastic changes are needed for nursing staff to reorganise and to take the patient's perspective into account and promote patient participation.

## 6 Limitations and strengths

Even though Chen et al. (45) argue that nurses can change SCI rehabilitation using action research, we encountered difficulties changing nursing rehabilitation in the direction of more patient participation. Limitations to our study may include that only a relatively small number of nursing staff (the eight co-researchers) actively participated in the study instead of the entire nursing staff. Furthermore, some challenges might have been avoided if inter-professional colleagues had been involved to a greater extent. We did not experience any difference in the engagement or ability to reflect between the group of registered nurses and the certified healthcare workers. However, there may be local variations in the length and content of the certified healthcare workers' education and qualifications worldwide.

The analysis is based on a large empirical material. Despite the local origin of action research which may not be directly referable and transferred to other settings (32) the in-depth analysis (34, 35) lead to a deeper understanding which strengthened the rigor and trustworthiness.

The strength in this study was that scientific knowledge was developed together with the nursing staff during continuous circles of reflections and dialogues combined with action. Thus, learning and acting were interrelated central aspects involving an understanding of the terms of meaning and value under which it was practiced (24). The nursing staff, with their profound knowledge about the organization, the specialty of SCI rehabilitation and their engagement in improving patient participation, tested and retested actions experiencing the challenges and obstacles. This involves four phases of knowledge development over a two-year time span. The depth of the nursing staff's work together with the analyses using the interpretive theory of Ricoeur provides us with local knowledge that can say something about a more general obstacle for nurses to improve patient participation in SCI-rehabilitation.

## 7 Conclusion

The patient's perspective in rehabilitation was considered essential and a cornerstone of patient participation in rehabilitation. This recognition made nursing staff strive to find time and inner calmness to be attentive listeners while working within the confines of task-oriented structures. They emphasise the need to change practice in rehabilitation and to provide conditions and an organisational structure that promote and incorporate caring and conversations with patients as equally important to other elements of the patient's rehabilitation.

## Data Availability

The raw data supporting the conclusions of this article will be made available by the authors, without undue reservation.

## Supplementary material

Supplementary Table S1 Co-researcher characteristics.
