# Supplementary figures and images for: Striving to establish patient participation in rehabilitation: the challenges experienced by nursing staff when changing practice to include the patient's perspective
Source: Front Rehabil Sci. 2024 Dec 19;5:1504984. doi: 10.3389/fresc.2024.1504984 (PMC11693675; doi:10.3389/fresc.2024.1504984)

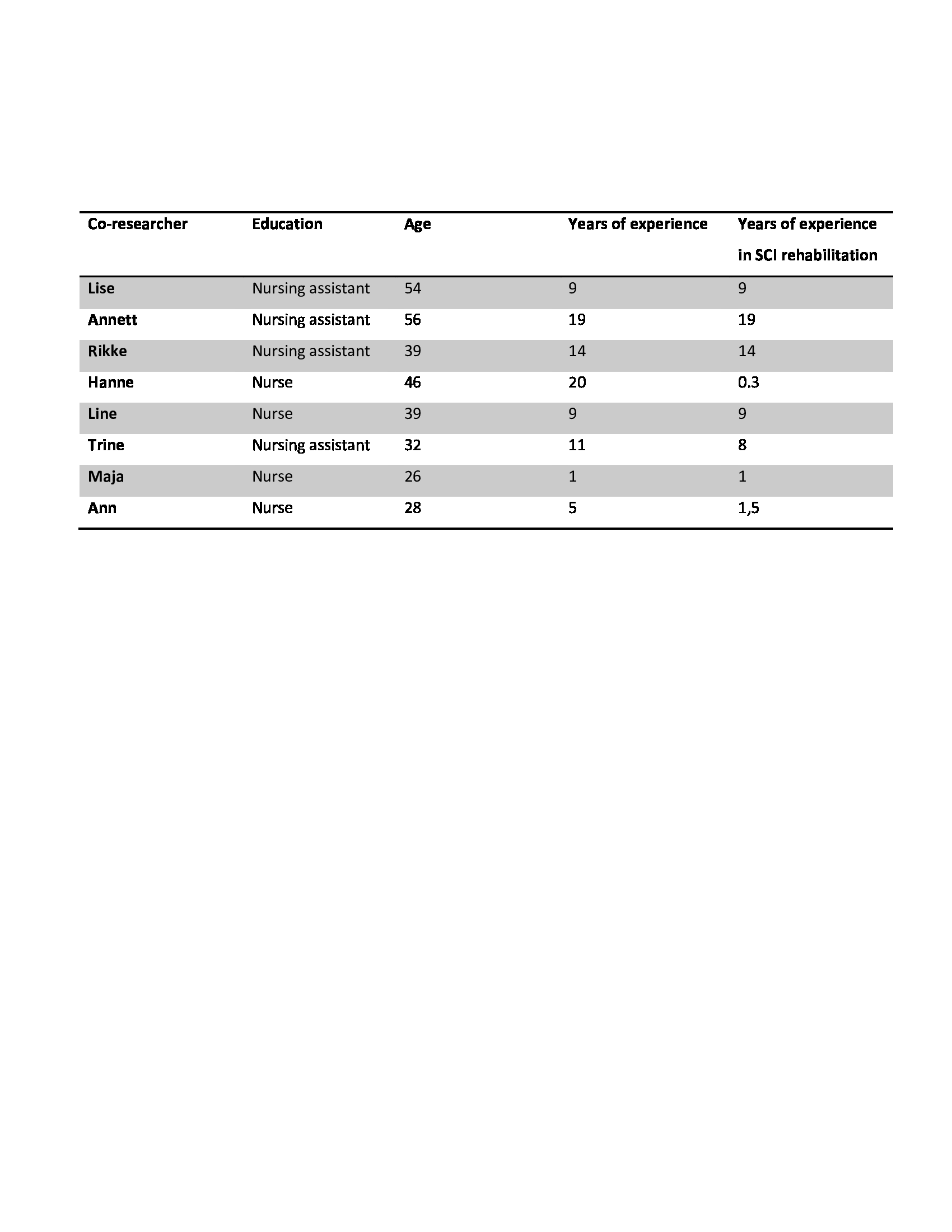

Supplement: Supplementary Table S1 — Co-researcher characteristics. [file Image1.png]
